# Influence of post-disaster evacuation on incidence of hyperuricemia in residents of Fukushima Prefecture: the Fukushima Health Management Survey

**DOI:** 10.1007/s10157-020-01924-6

**Published:** 2020-07-26

**Authors:** Shigeatsu Hashimoto, Masato Nagai, Tetsuya Ohira, Shingo Fukuma, Mitsuaki Hosoya, Seiji Yasumura, Hiroaki Satoh, Hitoshi Suzuki, Akira Sakai, Akira Ohtsuru, Yukihiko Kawasaki, Atsushi Takahashi, Kanako Okazaki, Gen Kobashi, Kenji Kamiya, Shunichi Yamashita, Shun-ichi Fukuhara, Hitoshi Ohto, the Fukushima Health Management Survey Group, Hitoshi Ohto, Masafumi Abe, Shunichi Yamashita, Kenii Kamiya, Seiji Yasumura, Mitsuaki Hosoya, Akira Ohtsuru, Akira Sakai, Shinichi Suzuki, Hirooki Yabe, Masaharu Maeda, Shirou Matsui, Keiya Fujimori, Tetsuo Ishikawa, Tetsuya Ohira, Tsuyoshi Watanabe, Hiroaki Satoh, Hitoshi Suzuki, Yukihiko Kawasaki, Atsushi Takahashi, Kotaro Ozasa, Gen Kobashi, Shigeatsu Hashimoto, Satoru Suzuki, Toshihiko Fukushima, Sanae Midorikawa, Hiromi Shimura, Hirofumi Mashiko, Aya Goto, Kenneth Eric Nollet, Shinichi Niwa, Hideto Takahashi, Yoshisada Shibata

**Affiliations:** 1 Radiation Medical Science Center, Fukushima Health Management Survey, Fukushima Medical University, Fukushima, Japan; 2 Departmnt of Endocrinology, Metabolism, Diabetology and Nephrology, Fukushima Medical University Aizu Medical Center, 21-2, Maeda, Tanisawa Kawahigashi, Aizuwakamatsu, Fukushima, 969-3492 Japan; 3 Department of Epidemiology, Fukushima Medical University School of Medicine, Fukushima, Japan; 4 Department of Healthcare Epidemiology, Kyoto University School of Public Health, Kyoto, Japan; 5 Center for Innovative Research for Communities and Clinical Excellence (CIRC2LE), Fukushima Medical University, Fukushima, Japan; 6 Department of Pediatrics, Fukushima Medical University School of Medicine, Fukushima, Japan; 7 Department of Public Health, Fukushima Medical University School of Medicine, Fukushima, Japan; 8 Department of Metabolism and Endocrinology, Juntendo University Graduate School of Medicine, Tokyo, Japan; 9 Department of Cardiology, Fukushima Medical University School of Medicine, Fukushima, Japan; 10 Department of Radiation Life Sciences, Fukushima Medical University School of Medicine, Fukushima, Japan; 11 Department of Radiation Health Management, Fukushima Medical University School of Medicine, Fukushima, Japan; 12 Department of Gastroenterology, Fukushima Medical University School of Medicine, Fukushima, Japan; 13 Department of Public Health, Dokkyo Medical University School of Medicine, Tochigi, Japan; 14 Research Institute for Radiation Biology and Medicine, Hiroshima University, Hiroshima, Japan; 15 Atomic Bomb Disease Institute, Nagasaki University, Nagasaki, Japan

**Keywords:** Hyperuricemia, Disaster, Evacuation, And life style

## Abstract

**Aim:**

After the Great East Japan Earthquake, over 160,000 residents in Fukushima Prefecture were forced to evacuate the area around the Fukushima Daiichi power plant following nuclear accident there. Health problems in these evacuees have since become a major issue. We have examined the association between evacuation and incidence of hyperuricemia among residents in Fukushima.

**Methods:**

We conducted a cohort study of residents aged 40–90 years without hyperuricemia at the time of the Fukushima disaster. Among 8173 residents who met the inclusion criteria before the disaster, 4789 residents (men: 1971, women: 2818; follow-up duration: 1.38 years; and follow-up rate: 58.6%) remained available for follow-up examinations at the end of March 2013. The main endpoint was incidence of hyperuricemia, defined by the Japanese committee guidelines, using local health data from before and after the disaster. We divided participants by evacuation status and compared outcomes between groups. Using a logistic regression model, we estimated the odds ratio for incidence of hyperuricemia, adjusting for potential confounders, age, gender, waist circumference, physical activity, and alcohol consumption.

**Results:**

Incidence of hyperuricemia was higher in evacuees (men 10.1%; women 1.1%) than in non-evacuees (men 7.4%, women 1.0%). Evacuees had higher body mass index, waist circumference, triglycerides, LDL-cholesterol, fasting plasma glucose, HbA1c, and lower HDL-cholesterol after the disaster than non-evacuees. We found that evacuation was associated with incidence of hyperuricemia (adjusted odds ratio: 1.38; 95% confidence interval: 1.03–1.86).

**Conclusion:**

This is the first study to demonstrate an association between evacuation after a disaster and increased incidence of hyperuricemia.

## Introduction

**Fig. 1:**
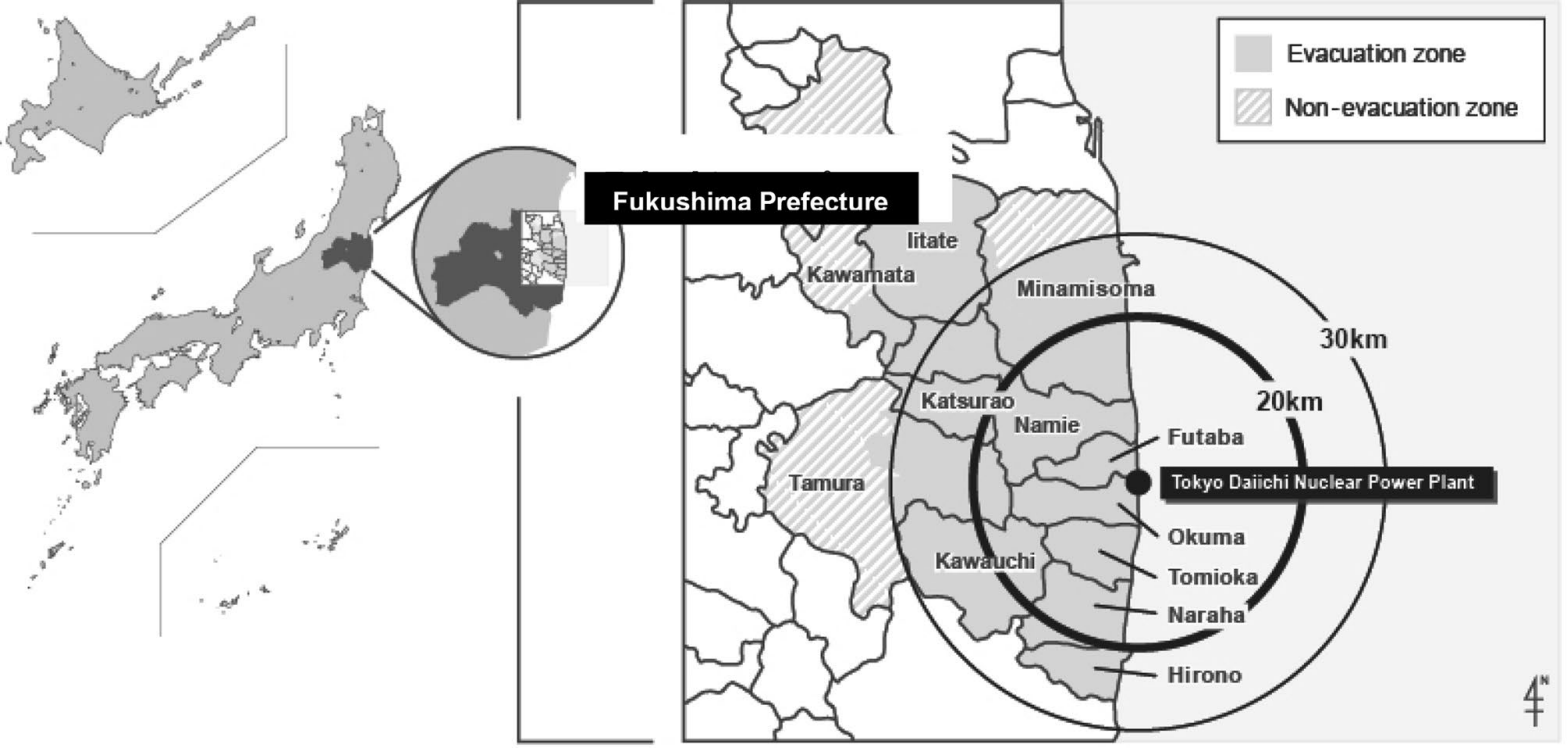
Geography of the evacuation zone, non-evacuation zone, and the Fukushima Daiichi nuclear power plant

When the Great East Japan Earthquake struck at a magnitude of 9.0 on March 11, 2011, a devastating tsunami and the Fukushima Daiichi nuclear power plant accident followed soon afterward. About 146,000 residents in a designated evacuation area around the nuclear power plant (Fig. 1) were subsequently forced to evacuate their homes.

In natural disasters like the Great East Japan Earthquake, there have been reports of an increased incidence of cardiovascular disease [1, 2] in the month directly following the onset of the event. These reveal that a disaster has a major influence on the development of cardiovascular diseases relatively soon afterward. However, the long-term effects of disasters on these health conditions have not been fully understood.

Long-term forced evacuation from the area surrounding Fukushima Daiichi may have forced changes in lifestyle for those affected, including altered diet, exercise habits, smoking status, and alcohol consumption. Since the triple disaster, health problems have become a major issue, especially among evacuees. It has been revealed that the incidence of lifestyle-related diseases associated with obesity, including hypertension [3] and metabolic syndrome, [4] is higher among evacuees than in non-evacuees. A link has been established between hyperuricemia incidence and these lifestyle-related diseases, and there has also been an association with morbidities [5, 6] and mortality [7]. However, very few reports have been published concerning the influence of post-disaster evacuation on hyperuricemia.

To estimate the influence of a change in living environment on the health status of residents following evacuation from a disaster area, we investigated the association between evacuation after the 2011 Great East Japan Earthquake and the incidence of hyperuricemia among residents in Fukushima Prefecture, Japan.

## Materials and methods

### Study population and design

Participants in this study were living in municipalities within 30 km of the Fukushima Daiichi Nuclear Power Plant at the time of the 2011 Great East Japan Earthquake. The townships involved were Hirono-machi, Tomioka-machi, Kawauchi-mura, Katsurao-mura, and Iitate-mura. The 2010 census recorded populations in these communities of 5418, 16,001, 2820, 1531, and 6209, respectively (total 31,979). Following the disaster, the government withdrew residents living within a 30 km radius of the nuclear power plant and in other nearby areas with high levels of radiation exposure (> 20 mSv/years). As a result, all residents of these two towns and three villages were forced to evacuate their homes in March 2011. To compare these evacuation areas, Tadami-machi (pop. 4932) and Minamiaizu-machi (pop. 17,864), which are some 150 km and 115 km away from Fukushima Daiichi power plant, respectively, were selected as a control area. With a combined population of 22,796 in 2010, neither town was directly affected by the disaster nor did they see any rise in air dose rates.

Health authorities have been offering residents aged ≥ 40 years in all these municipalities annual health checkups since 2008 through national health insurance, in an attempt to address metabolic syndrome. All analyses in this study are limited to residents aged 40–90 years. Between 2008 and 2010, 8173 residents (mean age: 64.3 years) from these areas participated in these health checkups.

Detailed methods of the Comprehensive Health Check (CHC) and the Fukushima Health Management Survey (FHMS) have been described previously [6, 8]. Follow-up examinations were conducted from June 2011 to the end of March 2013 as part of the CHC. Residents with missing data for plasma uric acid (UA) before the disaster (*n *= 1298) and after the disaster (*n *= 156) were excluded, as were those who were aged < 40 years at the time of the disaster (*n *= 55) and/or had hyperuricemia (> 7.0 mg/dL) before the disaster (*n *= 614). Others were removed due to their exercise habits (*n *= 1289), physical activities (*n *= 3), change in body weight (*n *= 4), smoking habit (*n *= 15), and alcohol intake (*n *= 4). This left 4789 residents (1971 men and 2818 women; follow-up rate: 58.6%) with complete anthropometric and laboratory results before and after the disaster (mean follow-up time: 1.38 years), who were analyzed in the present study. For analyses, we used data from the most recent period before the earthquake (2008–2010) and from the earliest year after 2011–2012 to define changes in health status before and after the disaster.

### Definitions and data collection

We defined hyperuricemia as a serum UA level more than 7.0 mg/dL, according to the definition of the Japanese committee for establishing diagnostic criteria for hyperuricemia [7].

During clinical examination, trained observers evaluated blood pressure and took anthropometric measurements using standard protocols and techniques. Participants were advised to avoid smoking, alcohol, caffeinated beverages, and exercise for at least 30 min before their blood pressure measurement. Body weight and height were also measured during these examinations. Weight was measured with subjects wearing light indoor clothing without shoes, and the subjects were barefoot when their heights were recorded. Waist circumference (WC) was measured above the navel at minimal respiration.

Various patient characteristics were examined under overnight fasting conditions, including height, weight, WC, body mass index (BMI), and blood pressure, as well as levels of aspartate aminotransferase, alanine aminotransferase, triglyceride (TG), high-density lipoprotein cholesterol (HDL-C), low-density lipoprotein cholesterol (LDL-C), HbA1c, fasting plasma glucose (FPG), urine protein, and urine sugar. Additional measurements included serum creatinine, hematocrit, and hemoglobin levels, as well as estimated glomerular filtration rate, UA levels, urinary testing for occult blood, and peripheral blood counts. These latter tests evaluated the number of red blood cells, platelets, and white blood cells. An interviewer asked residents about changes in body weight of 3 kg or more during the previous year, as well as exercise habits, sleeping quality, smoking status, and weekly alcohol intake. Participants who consumed ≥ 44 g ethanol or more per day were classified as being current excessive drinkers, after a previous study reported that increase in risk of all causes of mortality or coronary risk was observed among current excessive drinkers.

### Statistical analysis

The participants were divided into two groups: evacuees (*n *= 2298) and non-evacuees (*n *= 2491). Changes in body weight and BMI, and the proportion who were overweight before and after the disaster, were compared between the two groups using Student’s *t* test or a Wilcoxon rank-sum test. We used logistic regression analyses to estimate odds ratios (ORs) and 95% confidence intervals (CIs) for the association between evacuation and incidence of metabolic syndrome, after adjusting for potential confounders. The logistic regression model included the following variables: age (continuous), evacuation status (evacuee or non-evacuee), waist circumference (continuous), walking at least ≥ 1 h/day (yes or no), smoking status (current, never, or former), and alcohol consumption (current at ≥ 44 g/day, current at < 44 g/day, never, or former). Following our initial analyses, we repeated these after stratification by gender.

SAS version 9.4 (SAS Institute, Cary, NC, USA) was used for the analyses. All probability values for statistical tests were two-tailed, and *P* < 0.05 was regarded as statistically significant.

## Results

### Clinical characteristics before and after the disaster by evacuation status

The baseline clinical characteristics of the 4789 participants (1971 men and 2818 women) are listed in Table 1. Among men, plasma UA was lower at baseline in the evacuee group than in the non-evacuee group, while it was higher after the disaster in the evacuee group than in the non-evacuee group. On the other hand, among women, serum UA before the disaster and also change of serum UA levels after disaster were found to show no significant difference between evacuees and non-evacuees. Plasma creatinine at baseline among both genders was lower in the evacuee group than in the non-evacuee group, and decreased in both groups after the disaster. The decrease of plasma creatinine levels was greater in the evacuee group than in the non-evacuee group. TG levels at baseline among both genders were higher in the evacuee group than in the non-evacuee group, and increases in TG levels were higher in the evacuee group than in the non-evacuee group. HDL-C levels at baseline among both genders were lower in the evacuee group than in the non-evacuee group, and were a greater decrease in the evacuee group than in the non-evacuee group. LDL-C levels at baseline among women were higher in the evacuee group than in the non-evacuee group, but not among men, and LDL-C levels among both genders were a higher increase in the evacuee group than in the non-evacuee group after the disaster. FPG levels at baseline among women were lower in the evacuee group than in the non-evacuee group. FPG levels among both genders after the disaster were a greater increase in the evacuee group than in the non-evacuee group. HbA1c levels at baseline among both genders were higher in the evacuee group than in the non-evacuee group and did not change after the disaster.

**Table 1:** Characteristics of participants before and after the Great East Japan Earthquake

	Men				Women			
	Non-evacuee		Evacuee		Non-evacuee		Evacuee	
	Before	After	Before	After	Before	After	Before	After
Particitants, *n*	1002		969		1489		1329	
Age, years	65.4 (8.1)	66.7 (8.0)	63.7 (8.2)	65.2 (8.1)	65.1 (7.8)	66.4 (7.8)	63.0 (8.2)	64.4 (8.2)
Height, cm	163.0 (6.2)	162.9 (6.2)	163.3 (6.5)	163.0 (6.5)	150.5 (5.7)	150.3 (5.8)	151.1 (5.9)	150.9 (5.9)
Weight, kg	63.3 (9.0)	63.4 (9.2)	63.3 (9.7)	65.4 (9.6)	53.7 (8.2)	53.5 (8.1)	54.2 (8.4)	55.2 (8.6)
BMI^a^, kg/m^2^	23.8 (2.9)	23.8 (3.0)	23.7 (3.1)	24.6 (3.1)	23.7 (3.3)	23.7 (3.3)	23.7 (3.4)	24.3 (3.5)
Waist circumference, cm	84.9 (8.2)	85.0 (8.5)	84.8 (8.3)	86.7 (8.3)	84.0 (9.0)	84.3 (9.3)	84.7 (9.4)	84.9 (9.4)
SBP^b^, mmHg	135.9 (14.6)	134.9 (14.5)	134.1 (17.3)	133.3 (15.5)	133.1 (14.3)	132.5 (15.1)	130.8 (17.4)	130.2 (15.2)
DBP^c^, mmHg	81.6 (9.1)	81.2 (9.1)	79.3 (10.4)	79.2 (9.6)	79.7 (9.1)	78.8 (9.0)	76.3 (10.4)	77.1 (9.8)
Urate, mg/dL	5.5 (1.0)	5.6 (1.1)	5.4 (1.0)	5.6 (1.2)	4.6 (1.0)	4.6 (1.0)	4.5 (1.0)	4.5 (1.0)
Creatinine, mg/dL	0.8 (0.1)	0.8 (0.2)	0.8 (0.1)	0.8 (0.2)	0.7 (0.1)	0.7 (0.1)	0.7 (0.1)	0.7 (0.1)
Triglyceride, mg/dL	105.8 (72.9)	107.3 (78.2)	113.7 (99.8)	126.8 (80.2)	94.0 (50.0)	95.2 (51.5)	98.9 (56.2)	111.2 (63.8)
HDL-C^d^, mg/dL	59.4 (14.9)	58.8 (14.3)	57.0 (15.3)	54.2 (14.0)	65.4 (14.3)	65.2 (14.3)	62.5 (14.5)	61.0 (14.8)
LDL-C^e^, mg/dL	116.3 (28.9)	114.9 (28.3)	115.5 (29.5)	122.2 (30.6)	123.4 (27.4)	121.9 (27.2)	126.3 (30.6)	129.5 (32.7)
FPG^f^, mg/dL(25–75%)	96 (89–106)	98 (91–107)	95 (89–106)	99 (93–111)	93 (87–99)	93 (88–101)	91 (86–98)	95 (89–103)
HbA1c, %	4.4 (2.0)	4.4 (2.0)	5.6 (0.8)	5.6 (0.9)	4.4 (2.0)	4.4 (2.0)	5.5 (0.6)	5.5 (0.7)
3 kg weight change during 1 year, %	22.0	23.5	21.7	41.3	22.6	21.0	24.1	41.5
Exercise habit, %	32.1	33.5	34.7	32.8	26.1	24.1	30.3	31.4
Physical activity, %	43.4	43.2	41.9	39.2	35.7	33.7	37.0	34.9
Good sleep, %	79.2	81.8	78.5	72.0	77.0	77.5	72.2	59.0
Never or past smoker, %	74.0	77.5	69.4	72.3	95.6	96.2	94.1	94.6
Never drinker , %	14.5	18.6	17.3	20.3	38.4	44.9	43.4	50.5
Current drinker < 44 g/day, %	71.8	69.7	70.0	68.1	61.4	54.7	56.0	48.9
Current drinker ≥ 44 g/day, %	13.8	11.7	12.7	11.6	0.3	0.5	0.6	0.6

All values in parentheses, except for FPG, represent the standard deviation

^a^Body mass index, ^b^systolic blood pressure, ^c^diastolic blood pressure, ^d^high-density-lipoprotein-cholesterol, ^e^low--density-lipoprotein-cholesterol, ^f^fasting plasma glucose, ^g^physical activity (walking at least 1 h/day)

An anthropometric examination of men found no difference in body weight, BMI, and waist circumference at baseline between the evacuee and non-evacuee groups, though these parameters were subsequently found to increase more in the evacuee group than in the non-evacuee group. Among women, these parameters at baseline showed no difference between the groups either, but after the disaster, body weight and BMI increased in the evacuee group and conversely decreased in the non-evacuee group. Waist circumference in both groups increased after the disaster, and we observed a greater increase in the evacuee group than in the non-evacuee group.

The evacuee group had a higher proportion of marked body weight change (≥ 3 kg/year) in the year before baseline than the non-evacuee group. In addition, while the proportion of subjects reporting good sleeping quality decreased across both groups, the reduction was more marked among evacuees than non-evacuees.

### Impact of evacuation on incidence of hyperuricemia after the disaster (Table 2)

Crude incidence of hyperuricemia in both the overall population and in men was higher in the evacuee group [total: 4.9% (113/2298); men: 10.1% (98/969); and women: 1.1% (15/1329)] than in the non-evacuee group [total: 3.6% (89/2491); men: 7.4% (74/1002); and women: 1.0% (15/1489)]. Among women, it was not significant (Table 2).

**Table 2:** Association between evacuation status and incidence of hyperuricemia.

	*n*	Hyperuricemia	ORs (95% CIs)		
			Crude	Sex-age-adjusted	Multivariable adjusted^a^
All					
Evacuee	2298	113	1.40 (1.05–1.85)	1.37 (1.02–1.83)	1.38 (1.03–1.86)
Non-evacuee	2491	89	Reference	Reference	Reference
Men					
Evacuee	969	98	1.41 (1.03–1.94)	1.41 (1.03–1.93)	1.46 (1.06–2.02)
Non-evacuee	1002	74	Reference	Reference	Reference
Women					
Evacuee	1329	15	1.12 (0.55–2.30)	1.20 (0.58–2.47)	0.98(0.45–2.12)
Non-evacuee	1489	15	Reference	Reference	Reference

*OR* odds ratio, *CI* confidence interval

^a^Age (continuous), evacuation status (evacuee or non-evacuee), waist circumference (continuous), physical activity ;walking at least ≧1 h/day (yes or no), smoking status (current, never or former), and alcohol consumption (current at  ≧ 44 g/day, current at  < 44 g/day, never or former)

Logistic regression analysis revealed that evacuation was associated with higher incidence of hyperuricemia, even after adjusting for potential confounders of age, baseline clinical characteristics, and lifestyle [adjusted OR (95% CI): 1.38 (1.03–1.86); Table 2]. The association was essentially unchanged after further adjustment for eGFR at baseline (Table 3). We performed an additional subgroup analysis by gender and found that the association between evacuation and incidence of hyperuricemia was somewhat greater in men than in women [sex–age-adjusted OR (95% CI) in men: 1.41 (1.03–1.93); in women: 1.20 (0.58–2.47); Table 2].

**Table 3:** Association between evacuation status and incidence of hyperuricemia adjusted by eGFR

	*n*	Hyperuricemia	ORs (95%CIs) Multivariable adjusted^a^
All			
Evacuee	1449	75	1.54 (1.10–2.14)
Non-evacuee	2491	89	Reference
Men			
Evacuee	592	63	1.60 (1.11–2.30)
Non-evacuee	1002	74	Reference
Women			
Evacuee	857	12	1.24 (0.54–2.87)
Non-evacuee	1489	15	Reference

*OR* odds ratio, *CI* confidence interval

^a^Age (continuous), evacuation status (evacuee or non-evacuee), waist circumference (continuous), physical activity; walking at least  ≧ 1 h/day (yes or no), smoking status (current, never or former), alcohol consumption (current at  ≧ 44 g/day, current at  < 44 g/day, never or former), and eGFR (continuous)

## Discussion

To our best knowledge, this is the first longitudinal study with control to investigate the influence of the evacuation on the incidence of hyperuricemia following the Fukushima nuclear power plant accident, with data from 2008 to 2014. This study revealed an association between evacuee status and incidence of hyperuricemia, and evacuation as an independent risk factor for this incidence.

Evacuees may be susceptible to a number of cardiovascular risk factors, such as hypertension [4], impaired glucose tolerance/diabetes mellitus [5], and metabolic syndrome dyslipidemia [6], due to changes in lifestyle during long-term evacuation and insufficient food supply in the wake of the disaster. However, few studies have examined the acute or long-term effects of evacuation after a disaster on hyperuricemia caused by lifestyle changes. Evacuation forced the evacuees to change their eating habits, living environment, and climate, making it difficult for them to maintain their previous lifestyles due to displacement, their non-working status, or loss of job, as well as sudden disruption to their social networks after evacuation. The FHMS reports also associate the development of lifestyle-related diseases with increases in body weight without exception [8]. These lifestyle-related diseases are well-known risk factors for hyperuricemia. These previous reports support the present findings of an association between evacuation and incidence of hyperuricemia.

However, potential mechanisms of influence from evacuation on the development of hyperuricemia remain unclear, since the present study did not evaluate detailed lifestyle changes in food intake and social activity before and after the disaster. No clear trend was observed in the study of evacuees’ exercise habits: Physical activity of at least 30 min a day, two times or more a week, did not change in men, but did increase in women. Conversely, evacuees of both genders who would walk for 1 h or more per day saw the time they spent walking decreases after the disaster. Never smoker and never drinker labels increased after the disaster, as did the rate of sleep disturbance.

A recent study of a mental health and lifestyle survey by the FHMS showed reduced physical activity [9, 11] and greater alcohol consumption, psychological stress, and sleeping disturbance after the disaster among evacuees [11, 12]. This survey also showed that fear of radioactive fallout from the nuclear power plant had led to chronic anxiety and/or trauma [10–13]. Psychiatric disorders may have increased the prevalence in evacuees of sleep disorders, which have been associated with some risk factors of hyperuricemia, including obesity [12, 14], metabolic syndrome [13, 15], and hypertension [14, 16].

Gender subgroup analysis showed an association between evacuation and incidence of hyperuricemia among men, though not women. Although the mechanisms of gender variation are yet to be fully understood, there may be an association with an excessive increase in body weight, one of the risk factors for hyperuricemia, among the evacuees, especially among men [3]. Furthermore, the incidence of hyperuricemia among women was small (*n *= 30), compared with that among men (*n *= 172). This may take results among women toward the null.

Women evacuees saw a smaller increase in the proportion of never/past smoker than women non-evacuees after the disaster in this study. This is consistent with the result of the 2014 National Health and Nutrition Survey, which reported that the proportion of smokers in Fukushima Prefecture increased after the disaster from 23% in 2010 to 39.7% in 2012 [15, 17]. Previous studies reported an increase in smoking prevalence due to relapse and associated stress after the Canterbury earthquake in 2010 [16, 18]. Consequently, stress induced by a disaster may also be indirectly associated with hyperuricemia incidence due to an increase in smoking following an earthquake. Taken together, these previous findings suggest that positive changes in lifestyle after a disaster may be an important factor in mediating the influence of evacuation on hyperuricemia and preventing cerebrovascular or cardiovascular events.

The present study has a number of strengths, namely the inclusion of a relatively large number of residents from the evacuation zone, with residents of areas far from it as a control. Its assessment of plasma UA and hyperuricemia status before and after the disaster is also a strength. Doing so has allowed longitudinal analyses to identify an association between evacuation and risk of hyperuricemia. However, several potential limitations of this study should be considered. First, we did not evaluate socioeconomic status, which may influence several parameters associated with hyperuricemia. Second, the mean follow-up time of 1.33 years may not have been sufficient to examine long-term effects of evacuation on health outcomes. Third, the definition of hyperuricemia did not include the usage of urate-lowering drugs due to a lack of information about them. However, we consider that evacuees are more likely to use this drug than non-evacuees, since the evacuees had been offered free medical care, so the present result has been underestimated due to this bias. If we could consider the usage of urate-lowering drugs, the impact of evacuation on hyperuricemia would show more significantly. Finally, we could not obtain data on the usage of diuretics for the present study, though diuretics use is a well-known factor for the increase in serum UA levels. However, we believe that the present results have not been affected by this because the prevalence of diuretics may not be associated with evacuation status at baseline and would be small in the target population.

In conclusion, evacuation was associated with an increased incidence of hyperuricemia in Fukushima Prefecture after the 2011 Great East Japan Earthquake. Given this observed connection between evacuee status and hyperuricemia, we may conclude that these residents are at increased risk of cardiovascular events and chronic kidney disease (CKD), since previous epidemiological studies have reported that hyperuricemia is a predictive factor for not only cardiovascular diseases but also CKD and end-stage renal disease [5, 6, 19, 20]. Continuous health status surveillance among evacuees is necessary to reduce the increasing incidence of lifestyle-related diseases caused by long-term evacuation. The present study is the first to examine the influence of evacuation on incidence of hyperuricemia, and our findings may aid physicians in protecting the health of evacuees. We believe these data derived from the triple disaster in Fukushima will be called on to guard against similar effects in future disasters.
